# A Phase I/II Open-Label Multicenter Single-Arm Study of FABLOx (Metronomic 5-Fluorouracil Plus *nab*-Paclitaxel, Bevacizumab, Leucovorin, and Oxaliplatin) in Patients with Metastatic Pancreatic Cancer

**DOI:** 10.1089/pancan.2019.0012

**Published:** 2019-09-25

**Authors:** Vaibhav Sahai, M. Wasif Saif, Aparna Kalyan, Philip A. Philip, Caio M. Rocha-Lima, Allyson Ocean, Michael S. Ondovik, Diane M. Simeone, Sibabrata Banerjee, Rafia Bhore, Chrystal U. Louis, Vincent Picozzi

**Affiliations:** ^1^ Division of Hematology and Oncology, Department of Internal Medicine, University of Michigan, Ann Arbor, Michigan.; ^2^ Medical Oncology, Northwell Health Cancer Institute, Lake Success, New York.; ^3^ Department of Medicine, Northwestern University Feinberg School of Medicine, Chicago, Illinois.; ^4^ Department of Oncology, Karmanos Cancer Institute, Detroit, Michigan.; ^5^ Department of Oncology, Wake Forest School of Medicine, Bowman Gray Center, Winston-Salem, North Carolina.; ^6^ Department of Medical Oncology, Weill Cornell Medicine, New York, New York.; ^7^ Department of Medical Affairs, Celgene Corporation, Summit, New Jersey.; ^8^ Department of General Surgery, NYU Langone Perlmutter Cancer Center, New York, New York.; ^9^ Department of Oncology, Virginia Mason Medical Center, Seattle, Washington.

**Keywords:** 5-fluorouracil, metastatic pancreatic cancer, combination chemotherapy, *nab*-paclitaxel

## Abstract

**Purpose:** To evaluate safety and preliminary efficacy of metronomic 5-fluorouracil plus *nab*-paclitaxel, bevacizumab, leucovorin, and oxaliplatin (FABLOx) in patients with newly diagnosed metastatic pancreatic cancer (MPC).

**Methods:** A total of 12 treatment-naive patients (aged 18–65 years, Eastern Cooperative Oncology Group performance status [ECOG PS] ≤1) with MPC received 5-fluorouracil 180 mg/m^2^ per day (days 1–14 continuous infusion); *nab*-paclitaxel 75 mg/m^2^, leucovorin 20 mg/m^2^, and oxaliplatin 40 mg/m^2^ (days 1, 8, and 15); and bevacizumab 5 mg/kg (days 1 and 15) administered intravenously in each 28-day cycle. The primary end-point was incidence of dose-limiting toxicities (DLTs) in cycle 1. Safety was further evaluated as a secondary end-point; preliminary efficacy was also examined.

**Results:** Two DLTs (grade 3 anemia requiring transfusion and grade 3 mucositis unresponsive to treatment within 4 days of onset) were observed in one of six patients enrolled in dose cohort 1. Cohort 1 was expanded from 6 to 12 patients to further evaluate safety, per the investigators' recommendation. All patients discontinued treatment. The most common grade ≥3 adverse events were abdominal pain, fatigue, mucositis, and decreased neutrophil count. Objective response rate was 33% (four partial responses). Median progression-free survival (PFS) and overall survival (OS) were 5.6 (95% confidence interval [CI], 1.7–11.3) and 9.9 (95% CI, 4.4–13.2) months, respectively; 1-year PFS and OS rates were 12.2% (95% CI, 0.7–40.8) and 38.9% (95% CI, 12.6–65.0).

**Conclusion:** FABLOx is feasible and tolerable in patients newly diagnosed with MPC. However, preliminary efficacy data are inconclusive for continued investigation in a phase II trial.

## Introduction

Systemic chemotherapy remains the standard of care for patients with metastatic pancreatic cancer (MPC) despite the introduction of novel approaches, such as targeted therapy and immune checkpoint inhibitors, in other malignancies.^1,2^ In 1997, gemcitabine monotherapy was established as the standard of care in MPC, with subsequent trials using combination therapies with a gemcitabine-based backbone failing to demonstrate a clinically and statistically significant improvement in survival.^3–8^ In 2011, the PRODIGE 4/ACCORD 11 trial reported the FOLFIRINOX regimen (leucovorin, 5-fluorouracil [5-FU], irinotecan, and oxaliplatin), and, in 2013, the MPACT trial reported the combination of *nab*-paclitaxel plus gemcitabine. Each regimen demonstrated significantly longer overall survival (OS) than gemcitabine monotherapy in their respective phase III trials.^9–11^ In PRODIGE 4/ACCORD 11, the median OS was 11.1 months with FOLFIRINOX versus 6.8 months with gemcitabine monotherapy (HR, 0.57 [95% confidence interval (CI), 0.45–0.73]; *p* < 0.001).^9^ The MPACT trial demonstrated a median OS of 8.7 months with *nab*-paclitaxel plus gemcitabine versus 6.6 months with gemcitabine monotherapy (HR, 0.72 [95% CI, 0.62–0.83]; *p* < 0.001).^11^ On the basis of these results, either *nab*-paclitaxel plus gemcitabine or FOLFIRINOX are considered preferred regimens for the first-line treatment of patients with MPC.^2^

Although both regimens demonstrated a survival benefit, the patient's age, performance status and nutritional intake, and the regimen's distinct safety profiles are important considerations when selecting treatments for patients and designing prospective clinical trials. In the PRODIGE 4/ACCORD 11 trial, common grade 3/4 hematological adverse events associated with FOLFIRINOX were neutropenia (45.7%), thrombocytopenia (9.1%), anemia (7.8%), and febrile neutropenia (5.4%); common nonhematological adverse events were fatigue (23.6%), vomiting (14.5%), diarrhea (12.7%), sensory neuropathy (9.0%), elevated levels of alanine aminotransferase (7.3%), and thromboembolism (6.6%).^9^ In the MPACT trial, common grade ≥3 hematological adverse events associated with *nab*-paclitaxel plus gemcitabine were neutropenia (38%), leukopenia (31%), thrombocytopenia (13%), and anemia (13%); common nonhematological adverse events were fatigue (17%), peripheral neuropathy (17%), and diarrhea (6%).^10,11^

In an effort to mitigate safety concerns with high-dose intermittent chemotherapy while retaining efficacy of combination regimens, multiple strategies have been used, including metronomic dosing and alternating or in-tandem administration of *nab*-paclitaxel and FOLFIRINOX. Isacoff et al. evaluated low-dose metronomic chemotherapy with 5-FU, *nab*-paclitaxel, leucovorin, and oxaliplatin plus bevacizumab in 65 patients with advanced PC and reported the results in a retrospective analysis.^12^ Unfortunately, toxicity was still a concern with this regimen, and 22 patients discontinued treatment due to adverse events; however, efficacy was encouraging, with a median OS of 19 months and an objective response rate of 49%. Similarly, retrospective reports of other metronomic regimens, such as POLF (paclitaxel, oxaliplatin, leucovorin, and 5-FU) and PILF (paclitaxel, irinotecan, leucovorin, and 5-FU) support the continued investigation of low-dose metronomic regimens for the treatment of MPC.^13^

Additional combination therapies have been evaluated in single-arm studies of FOLFOX-A (leucovorin, 5-FU, oxaliplatin, and *nab*-paclitaxel)^14^ as well as the alternation or in-tandem administration of *nab*-paclitaxel and FOLFIRINOX in the GABRINOX, SEENA-1, and NabucCO studies, which all reported safety profiles comparable with those demonstrated in the MPACT and PRODIGE trials.^15–17^ These studies demonstrate that toxicity remains a concern with combination chemotherapy regimens despite promising efficacy.

In this study we report the phase I results of a multicenter single-arm study investigating the regimen of metronomic 5-FU plus *nab*-paclitaxel, bevacizumab, leucovorin, and oxaliplatin (FABLOx) in patients with newly diagnosed MPC. The oxaliplatin and *nab*-paclitaxel doses were lower than those administered in the study by Isacoff et al. in an effort to reduce toxicity while maintaining efficacy.^18^ Previous preclinical and clinical investigations have suggested that bevacizumab and other similar antiangiogenic therapies may prolong the antitumor effect of paclitaxel.^19,20^ The primary objective of this study was to determine dose-limiting toxicities (DLTs) of metronomic FABLOx in patients with newly diagnosed MPC.

## Methods

The study was approved by each participating site's institutional review board or independent ethics committee and was conducted in compliance with the International Council for Harmonisation Good Clinical Practice guidelines and with the general ethical principles of the Declaration of Helsinki. Written informed consent was obtained from all patients before study entry. This study was registered on ClinicalTrials.gov under NCT02620800.

### Patients

Owing to limited experience and the toxicities associated with this five-drug regimen, patient enrollment was limited to those with good performance status, limited comorbidities, and age ≤65 years. Patients (aged 18–65 years) with histologically or cytologically confirmed MPC, an Eastern Cooperative Oncology Group performance status (ECOG PS) of 1 or 0, and no prior systemic chemotherapy or investigational therapy (other than as a radiosensitizer) for PC were eligible. Adequate blood and organ function as well as measurable disease per Response Evaluation Criteria In Solid Tumors (RECIST) version 1.1 were required for inclusion.^21^ Patients were excluded if they had grade >1 peripheral neuropathy, a history of malignancy other than PC in the past 3 years, known brain metastases, or an allergy/hypersensitivity to any investigational products or any of their excipients.

### Study design

**FIG. 1.**
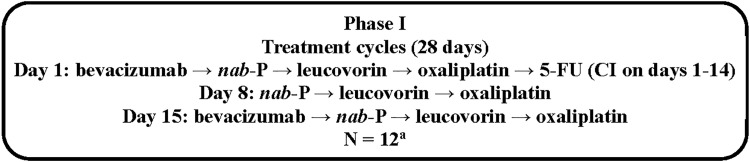
Study design for phase I. ^a^Initially, 6 patients were enrolled at dose level 1, but the cohort was expanded to 12 patients based on investigators' recommendation. 5-FU, 5-fluorouracil; CI, continuous infusion; DLT, dose-limiting toxicity; *nab*-P, *nab*-paclitaxel.

In this multicenter single-arm phase I dose de-escalation study, the regimen was given as follows: 5-FU 180 mg/m^2^ per day on days 1 to 14 through continuous infusion; *nab*-paclitaxel 75 mg/m^2^ intravenously (IV) on days 1, 8, and 15; bevacizumab 5 mg/kg IV on days 1 and 15; leucovorin 20 mg/m^2^ IV on days 1, 8, and 15; and oxaliplatin 40 mg/m^2^ IV on days 1, 8, and 15 in each 28-day treatment cycle (the order of receipt of agents is summarized in Fig. 1; the planned dose de-escalation protocol is summarized in Supplementary Table S1). As noted previously, the oxaliplatin and *nab*-paclitaxel doses were lower than those administered in the study by Isacoff et al. in an effort to potentially reduce toxicity.^18^ Patients were treated until disease progression, unacceptable toxicity, withdrawal of consent, physician decision, or death.

### Assessment of safety end-points and preliminary efficacy

The primary end-point was the incidence of DLTs, assessed in cycle 1 (DLTs are defined in Supplementary Table S2). A minimum of six patients were to be enrolled in each consecutive dosing cohort. If DLTs occurred in more than or equal to two of six patients, the dose would be de-escalated. Safety was continuously evaluated by incidence and severity of treatment-emergent adverse events (TEAEs) according to the Medical Dictionary for Regulatory Activities and the National Cancer Institute Common Terminology Criteria for Adverse Events version 4.03.

Progression-free survival (PFS) and OS were represented by Kaplan–Meier curves and median survival times with respective 95% CIs. After treatment discontinuation, each patient was followed up for disease progression and survival every 90 days for a minimum of 18 months. The objective response rate was assessed by computed tomography or magnetic resonance imaging according to RECIST v1.1 criteria; results are presented by frequency and percentages with 95% CIs reported for the proportion of responsive patients (complete or partial response) from the total number of patients evaluable for response by RECIST criteria.

### Statistical methods

A minimum of six patients could be enrolled in each consecutive dosing cohort, with enrollment of additional patients per the investigators' recommendation based on the totality of the data, to further evaluate any dose level. A total of 12–24 patients were planned for enrollment, depending on the number of dose levels examined and number of patients enrolled at each dose level.

## Results

### Patient disposition

A total of 19 patients were screened for inclusion, but 7 were excluded at screening (MPC not confirmed, unacceptable blood chemistry levels at screening, did not agree to participate in the study, pregnant/nursing [lactating] woman, or other significant medical condition, laboratory abnormality, or psychiatric illness that prevented participation in the study). The treated and response-evaluable populations consisted of 12 patients. All patients discontinued treatment, with the majority discontinuing due to progressive disease (*n* = 7) followed by adverse events (*n* = 4) and withdrawal by patient (*n* = 1).

### Baseline characteristics

Patient demographics and baseline characteristics are described in Table 1. The median age was 57.5 years and most patients (75.0%) were male. The most common sites of metastasis were the liver (83.3%), peritoneum (66.7%), and lung/thoracic cavity (41.7%).

**Table 1.** Baseline Demographics and Characteristics

Characteristic	*N* = 12
Age, median (range), years	57.5 (41–64)
Gender, *n* (%)	
Male	9 (75.0)
Female	3 (25.0)
Eastern Cooperative Oncology Group performance status, *n* (%)	
0	7 (58.3)
1	5 (41.7)
Site(s) of metastasis, *n* (%)	
Liver	10 (83.3)
Peritoneum	8 (66.7)
Lungs/mediastinum	5 (41.7)
Other	2 (16.7)
No. of metastatic sites	
1	4 (33.3)
2	3 (25.0)
3	5 (41.7)
>3	0

### Safety

Six patients were enrolled in the first dose cohort. One patient experienced two DLTs (grade 3 anemia requiring a transfusion and grade 3 mucositis unresponsive to medical treatment within 4 days of onset). The starting dose cohort was expanded from 6 to 12 patients to further evaluate the rates of mucositis, per the investigators' recommendation. No additional DLTs were observed in the expanded cohort. Dose de-escalation was not required.

A summary of TEAEs is provided in Table 2. The most common TEAEs of grade ≥3 were abdominal pain, fatigue, mucositis, and decreased neutrophil count. Sepsis was the only grade 4 TEAE (one patient, not suspected to be related to any study drug). One patient experienced a grade 3 serious adverse event of peritoneal infection that worsened to a grade 5 event. Seven patients experienced ≥1 serious TEAE.

**Table 2.** Safety

		*N* = 12
Patients with ≥1 grade ≥3 TEAE, *n* (%)		12 (100.0)
Patients with ≥1 serious TEAE, *n* (%)		7 (58.3)

AE, *n* (%)	Grade 1/2^a^	Grade ≥3 (occurring in ≥10% of patients)
Hematological AEs		
Neutrophil count decreased	0	3 (25.0)
Anemia	1 (8.3)	2 (16.7)
Nonhematological AEs		
Abdominal pain	5 (41.7)	3 (25.0)
Fatigue	8 (66.7)	3 (25.0)
Mucosal inflammation	3 (25.0)	3 (25.0)
Alanine aminotransferase increased	0	2 (16.7)
Blood alkaline phosphatase increased	1 (8.3)	2 (16.7)
Nausea	5 (41.7)	2 (16.7)
Peripheral sensory neuropathy	3 (25.0)	2 (16.7)
Stomatitis	4 (33.3)	2 (16.7)

^a^ Inclusion of grade 1/2 AEs are based on inclusion criteria for grade ≥3 AEs.

AE, adverse event; TEAE, treatment-emergent adverse event.

### Treatment exposure and dose modifications

All patients were treated at the starting dose level. Treatment exposure and dose modifications for each agent within the FABLOx regimen are summarized in Table 3. The median treatment duration was 27–28 weeks for all agents in the FABLOx regimen except oxaliplatin, for which the median treatment duration was 22 weeks.

**Table 3.** Treatment Exposure and Dose Modifications

	*N* = 12				
Parameter	Bevacizumab	*nab*-Paclitaxel	Leucovorin	Oxaliplatin	5-Fluorouracil
Treatment exposure					
Treatment duration, median (range), weeks	27.85 (3.7–51.4)	27.00 (3.7–51.4)	27.85 (3.7–51.4)	22.00 (3.7–51.4)	28.00 (3.9–53.6)
Treatment cycles, median (range), *n*	7.0 (1–13)	7.0 (1–13)	7.0 (1–13)	5.5 (1–13)	7.0 (1–13)
Relative dose intensity, median, %	94.02	88.25	93.64	88.41	84.35
Cumulative dose, median (range), mg	4685.50 (500.0–8217.0)	1343.63 (222.0–2737.9)	413.87 (57.9–728.9)	573.73 (115.8–1462.2)	14,253.36 (2161.6–25,630.9)
Dose modifications					
Patients with ≥1 dose reduction, *n* (%)	0	5 (41.7)	0	6 (50.0)	7 (58.3)
Patients with ≥1 dose delay, *n* (%)	5 (41.7)	5 (41.7)	5 (41.7)	5 (41.7)	5 (41.7)

### Efficacy

**FIG. 2.**
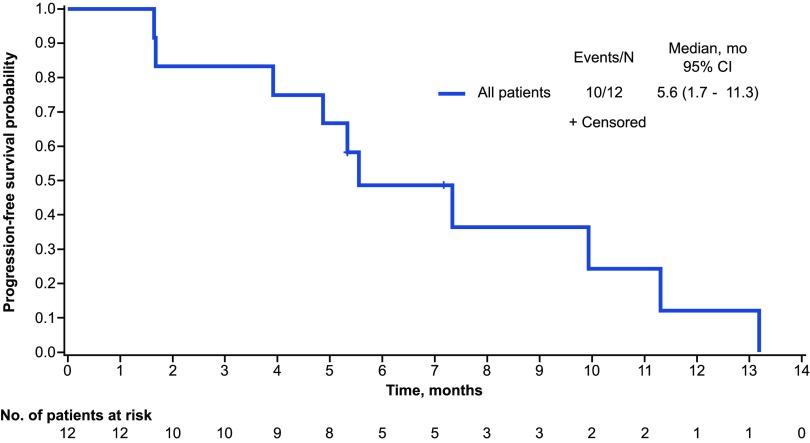
Progression-free survival.

**FIG. 3.**
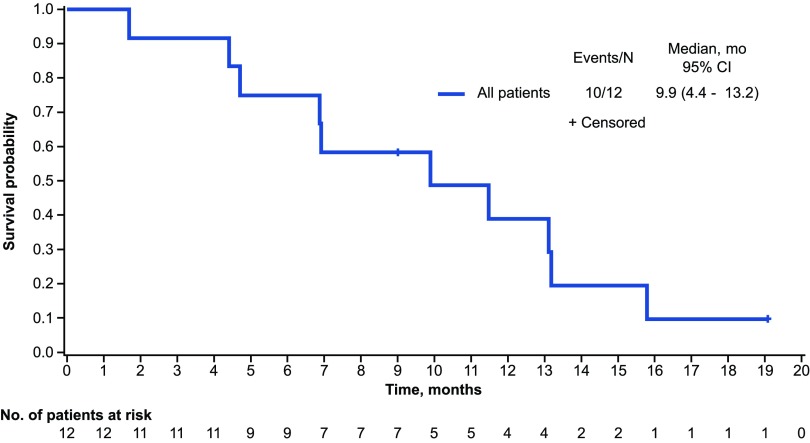
Overall survival.

The objective response rate (best response: complete, or partial response) was 33% and the disease control rate (complete response, partial response, or stable disease) was 92%; four patients achieved a partial response and seven had stable disease (Table 4). The median PFS was 5.6 months (95% CI, 1.7–11.3) and the 1-year PFS rate was 12.2% (95% CI, 0.7–40.8; Fig. 2). The median OS was 9.9 months (95% CI, 4.4–13.2) and the 1-year estimated OS rate was 38.9% (95% CI, 12.6–65.0%; Fig. 3).

**Table 4.** Response Rates

Outcome, *n* (%)	*N* = 12
Best overall response	
Complete response	0
Partial response	4 (33.3)
Stable disease^a^	7 (58.3)
Progressive disease	1 (8.3)
Objective response rate	4 (33.3)
95% confidence interval	13.8–60.9
Disease control rate^a^	11 (91.7)

^a^ Minimum time to stable disease was not specified in the protocol.

## Discussion

This phase I trial evaluated the safety of the FABLOx regimen in patients with MPC. Results from this study suggest that the FABLOx regimen is both feasible and tolerable in patients with MPC with an ECOG PS of 0 or 1, good organ function, and no prior systemic chemotherapy. The FABLOx regimen evaluated in this study demonstrated similar tolerability to the combination therapy regimen reported by Isacoff et al.^12^ In the Isacoff et al. study, 22 patients (34%) discontinued due to toxicity, which is similar to this study, in which four patients (33%) treated with FABLOx discontinued due to adverse events.^12^

Multidrug cytotoxic regimens have shown improvement in survival in patients with MPC compared with gemcitabine monotherapy but at the expense of increased toxicity.^9,10^ Multiple trials are evaluating alternative cytotoxic regimens in an attempt to further improve efficacy without increasing toxicity. In the single-arm study by Safran et al. at a single institution, the FOLFOX-A regimen resulted in a median survival of 15 months in patients with MPC and had a comparable toxicity profile to that of standard-of-care regimens.^14^ Similarly, in an attempt to reduce toxicity and the early development of chemotherapy resistance, a few trials have studied alternating gemcitabine- and 5-FU-based regimens. The phase I/II GABRINOX study investigated sequential monthly administration of *nab*-paclitaxel plus gemcitabine followed by FOLFIRINOX in patients with MPC. The median OS was reported to be 17.8 months, which was encouraging; however, the rates of grade 3/4 thrombocytopenia, neutropenia, and diarrhea were higher than expected.^9,10,15^ In the phase II SEENA-1 study, either *nab*-paclitaxel plus gemcitabine was followed by modified FOLFIRINOX (no bolus 5-FU), or *nab*-paclitaxel plus gemcitabine was given alternating with modified FOLFIRI (5-FU, leucovorin, and irinotecan) for up to 48 weeks.^17,22^ The efficacy results were modest (median OS, 12.3 months in all patients and 13.5 months in patients with disease control after 8 weeks of *nab*-paclitaxel plus gemcitabine), and the safety profile was generally similar to that in the MPACT and PRODIGE 4/ACCORD 11 trials, with common grade ≥3 toxicities, including neutropenia (43%), fatigue (22%), anemia (21%), and thrombocytopenia (15%).^9,10,17^ A single-institution extension of the SEENA-1 regimen of alternating *nab*-paclitaxel plus gemcitabine with FOLFIRI reported improved survival outcomes (median OS, 16.3 months).^22^ Recently, the phase II NabucCO study investigated the effect of modifying FOLFIRINOX by replacing either oxaliplatin (*nab*-FOLFIRI) or irinotecan (*nab*-FOLFOX) with *nab*-paclitaxel.^16^ These modified regimens demonstrated similar survival to that with FOLFIRINOX (median OS, 10.8–13.2 months) but with lower rates of neutropenia.

Overall, these studies examining *nab*-paclitaxel- and 5-FU-based regimens have demonstrated feasibility and relatively consistent survival outcomes; however, they are limited by their design (single-arm and single-institution studies). The survival results with FABLOx are similar to those with other prospectively evaluated combination regimens that have reported median OS values ranging from 7.3 to 13.2 months.^16,23,24^ The adverse event profile of FABLOx is also consistent with that reported in previous studies.^16,17,25^ This study supports the relatively consistent outcomes observed with the use of *nab*-paclitaxel- and 5-FU-based regimens in MPC treatment. Although treatment with FABLOx is feasible in patients with MPC, further investigation is not planned due to the lack of significant improvement in efficacy and the need for exigent 2-week continuous 5-FU infusion. These results further underscore the need for novel agents and greater insight into the biology of PC to understand how to better treat this patient population.

## Conclusion

Results from phase I of this study demonstrate a tolerable safety profile for metronomic FABLOx in patients with newly diagnosed MPC, but preliminary efficacy data are inconclusive for continued investigation in a phase II trial, given the small number of patients in the study.

## Supplementary Material

Supplemental data

## Abbreviations Used

5-FU: 5-fluorouracil
DLTs: dose-limiting toxicities
ECOG PS: Eastern Cooperative Oncology Group performance status
FABLOx: metronomic 5-fluorouracil plus *nab*-paclitaxel, bevacizumab, leucovorin, and oxaliplatin
IV: intravenously
MPC: metastatic pancreatic cancer
OS: overall survival
PFS: progression-free survival
PILF: paclitaxel, irinotecan, leucovorin, and 5-FU
POLF: paclitaxel, oxaliplatin, leucovorin, and 5-FU
RECIST: Response Evaluation Criteria In Solid Tumors
TEAE: treatment-emergent adverse event
