# Supplementary material for: A Phase I/II Open-Label Multicenter Single-Arm Study of FABLOx (Metronomic 5-Fluorouracil Plus nab-Paclitaxel, Bevacizumab, Leucovorin, and Oxaliplatin) in Patients with Metastatic Pancreatic Cancer
Source: J Pancreat Cancer. 2019 Sep 25;5(1):35–42. doi: 10.1089/pancan.2019.0012 (PMC6761587; doi:10.1089/pancan.2019.0012)
Supplement: Supplemental data [file Supp_TableS1-S2.pdf]

Supplementary Data

Supplementary Table S1. Planned Dose De-Escalation Protocol

Dose level	Leucovorin D1, 8, 15 (mg/m^2)	5-FU CI D1 to 14 (mg/m^2 per day)	Oxaliplatin D1, 8, 15 (mg/m^2)	*nab*-Paclitaxel D1, 8, 15 (mg/m^2)	Bevacizumab biweekly D1, 15 (mg/kg)
Start	20	180	40	75	5
−1	20	135	30	60	5
−2	20	90	20	50	5

5-FU, 5-fluorouracil; CI, continuous infusion; D, day.

Supplementary Table S2. Dose-Limiting Toxicity Definitions

DLT defined as ≥1 AE related to FABLOx during cycle 1
Any toxicity starting during the first 28 days of treatment and requiring ≥14 days of treatment interruption
Grade 3/4 neutropenia associated with fever >38.5°C (febrile neutropenia)
Grade 3 anemia requiring a transfusion
Any grade 4 hematological toxicity lasting >7 days
Grade 4 thrombocytopenia or grade 3 or 4 thrombocytopenia associated with clinically significant bleeding
Grade 3/4 nonhematological toxicity except for fatigue, attributable to any of the IPs and unresponsive to medical treatment within 4 days of onset
Patients who experience grade ≥3 hyperbilirubinemia as an apparent DLT will be tested for conjugated bilirubin levels to rule out Gilbert syndrome or other conditions causing elevated levels principally of unconjugated bilirubin. If a phase I patient experienced a grade ≥3 hyperbilirubinemia due principally to unconjugated bilirubin, it was not to be defined as a DLT. The patient was to be removed from the study and the phase I patient was to be replaced.
Grade ≥2 pneumonitis or interstitial lung disease

AE, adverse event; DLT, dose-limiting toxicity.
